# Editorial: Positive psychological interventions: How, when and why they work: Beyond WEIRD contexts

**DOI:** 10.3389/fpsyg.2022.1021539

**Published:** 2022-10-03

**Authors:** Wenjie Duan, Jeff Klibert, Marijke Schotanus-Dijkstra, Susana Llorens, Machteld van den Heuvel, Claude-Helene Mayer, Dan Tomasulo, Yujing Liao, Llewellyn Ellardus van Zyl

**Affiliations:** ^1^School of Social and Public Administration, East China University of Science and Technology, Shanghai, China; ^2^Department of Psychology, Georgia Southern University, Statesboro, GA, United States; ^3^Department of Positive Psychology and Technology, University of Twente, Enschede, Netherlands; ^4^WANT Research Team, Department of Developmental Psychology, Education, Social Psychology and Methodology, Universitat Jaume I, Castelló de la Plana, Spain; ^5^Faculty of Social and Behavioural Sciences Programme, Work and Organizational Psychology Group, University of Amsterdam, Amsterdam, Netherlands; ^6^Department of Industrial Psychology and People Management, University of Johannesburg, Johannesburg, South Africa; ^7^Department of Counseling and Clinical Psychology, Columbia University, New York, NY, United States; ^8^Department of Industrial Engineering and Innovation Sciences, University of Eindhoven, Eindhoven, Netherlands; ^9^Optentia Research Unit, North-West University, Vanderbijlpark, South Africa; ^10^Department of Human Resource Management, University of Twente, Enschede, Netherlands; ^11^Department of Social Psychology, Institut für Psychologie, Goethe University, Frankfurt am Main, Germany

**Keywords:** positive psychological interventions, positive interventions, positive coaching, positive psychology, personal development

## Introduction

Since the concept of “positive psychology” was put forward in 1998 (Seligman, 2002), the corresponding intervention field has also developed, effectively transforming theory into a dynamic set of pathways to support individual and community wellbeing efforts. Positive psychological interventions (PPIs) is the general term for a series of practical application activities designed to strengthen “positive resources” (van Zyl et al., 2017; Ng and Ong, 2022). PPIs offer a unique perspective in the applied psychological space (van Zyl and Rothmann, 2022). Traditionally, applications of applied psychology were designed to address deficits and manage symptoms to help individuals and communities rebound from adversity and recover from injury (Seligman and Csikszentmihalyi, 2000). However, such approaches are limiting; they characterize individuals and communities primarily through their deficits and symptoms (van Zyl et al., 2020a; Richter et al., 2021). To combat these limitations, PPIs are designed around the identification and utilization of personal strengths as a means to facilitate personal growth, community functioning and organizational thriving (van Zyl and Rothmann, 2021; van Zyl and Salanova, 2022).

These PPIs are shown to have significant short- and medium-term effects which increased their popularity in practice (Jorgensen-Graupner and van Zyl, 2019; Krifa et al., 2021; Van Zyl et al., 2021). Resultantly, an ever-increasing array of PPIs are developed and validated to facilitate positive emotional experiences (e.g., Quoidbach et al., 2015), character strengths (e.g., Chérif et al., 2021), and community functioning/wellbeing (e.g., Shankland and Rosset, 2017). Moreover, given the increased interest in developing wide arrays of PPIs, researchers are becoming increasingly interested in the effectiveness of these interventions in different contexts, communities and cultures (van Zyl et al., 2020b; Duan et al., 2022). Despite these gains, the field must continually move in a more inclusive and socially just direction to ensure all communities have access to effective, reliable, and culturally responsive PPIs (Pedrotti et al., 2021).

At the present, PPIs are criticized for being a Western-, Educated-, Industrialized-, Rich- and Democratic- (WEIRD) enterprise, neglecting under-privileged, under-represented, and under-served groups' experiences and expressions of strength, while also ignoring the cultural origins of the positive states, traits, and behaviors PPIs aim to improve (Hendriks et al., 2019; Stander and van Zyl, 2019; Worthington and Van Zyl, 2021). Lack of cultural representation in positive psychology is a well-established pattern within the literature (Donaldson et al., 2021; van Zyl and Rothmann, 2022). For instance, <1% (0.42%) of all strength-based and PPI articles include representation from Lesbian, Gay, Bisexual, Transgender, and Queer+ (LGBTQ+) individuals and communities (Vaughan et al., 2014). This trend is a microcosm of a bigger issue, where a recent bibliographic analysis reveals ~0.02% of randomized clinical trials on PPIs consider Non-WEIRD contexts (Hendriks et al., 2019). Moreover, effects highlighting the efficacy of some PPIs established through westernized and well-resourced countries are weaker when such PPIs are evaluated in under-served and culturally diverse communities across the world (e.g., Khanna and Singh, 2019).

Despite emerging guidelines regarding how PPIs can be adapted to different cultural contexts (Schick et al., 2021), research exploring the intersection between PPIs and non-WEIRD cultural identities is tentative, incomplete, and disjointed (Rao and Donaldson, 2015), leading others to question “If,” “When,” “How,” and “Why” PPIs work in non-WEIRD contexts. To address these questions, we collected a series of articles to provide more inclusive and socially just methods of evaluating PPIs in different cultural contexts. We believe such work will be critical in identifying unique insights into how PPIs can be framed, constructed, and evaluated to change the dialogue behind how positive psychological theory and applications can support culturally affirmative action across diverse geographic locations, lived experiences, values, and cultural identities. The collected works (*N* = 9) in this Research Topic are summarized in Table 1. Each work is allocated to a specific section depending upon the type of question being evaluated. Notably, the works address one of three questions: whether PPIs are effective in non-WEIRD contexts (IF)?, what are the diverse contexts by which PPIs work (WHEN)?, and what are the unique methodological factors and conditions required to ensure PPIs yield beneficial results in diverse samples (HOW/WHY)?

**Table 1:** Summary of contributions to the Research Topic.

**No**	**Author**	**Title**	**Purpose**	**Views**	**Citations**
1	Donaldson et al.	*Following the Science to Generate Well-Being: Using the Highest-Quality Experimental Evidence to Design Interventions*	While much of the wellbeing literature is based on descriptive and correlational studies, this paper evaluates a growing body of causal evidence from high-quality randomized controlled trials (RCTs) that test the efficacy of positive psychology interventions (PPIs). This systematic review analyzed the findings from 25 meta-analyses, 42 review papers, and the high-quality RCTs of PPIs designed to generate wellbeing that were included within those studies.	3,097	3
2	Beyebach et al.	*Bibliometric Differences Between WEIRD and Non-WEIRD Countries in the Outcome Research on Solution-Focused Brief Therapy*	The aim of this study was to examine the development of outcome research on SFBT and to determine whether it is predominantly carried out in Western, Educated, Industrialized, Rich and Democratic (WEIRD) countries.	2,426	3
3	Chamorro-Garrido et al.	*Autobiographical Memory, Gratitude, Forgiveness and Sense of Humor: An Intervention in Older Adults*	The objective of this study was to verify whether an intervention based on Autobiographical Memory, Forgiveness, Gratitude, and Sense of humor would increase quality of life in institutionalized older adults.	12,228	0
4	Torkhani et al.	*Improving Health of People With Multiple Sclerosis From a Multicenter Randomized Controlled Study in Parallel Groups: Preliminary Results on the Efficacy of a Mindfulness Intervention and Intention Implementation Associated With a Physical Activity Program*	The objective of this study is to investigate the efficacy of psychological Interventions—Mindfulness or Implementation Intention—associated with a Physical Activity program, delivered *via* internet, in reducing Multiple Sclerosis symptoms.	1,104	0
5	Calcagni et al.	*Differential Effects of Mindfulness-Based Intervention Programs at Work on Psychological Wellbeing and Work Engagement*	The purpose of the paper was to determine the effectiveness of two different mindfulness-based interventions to explore the differential effects on different facets of mindfulness, dimensions of psychological wellbeing, work engagement, performance, and stress of a participant.	1,941	1
6	Klibert et al.	*Savoring Interventions Increase Positive Emotions After a Social-Evaluative Hassle*	The purpose of the current research was to examine whether different savoring interventions could increase important coping resources (i.e., positive emotions) in response to a social-evaluative hassle.	952	0
7	Basurrah et al.	*Positive Psychology Interventions as an Opportunity in Arab Countries to Promoting Well-Being*	This opinion paper aimed to explore the opportunities for positive psychological intervention in Arab countries. Specifically, it reflected upon the importance of positive psychological approaches to facilitate wellbeing in these contexts	1,195	0
8	Botha et al.	*Flourishing Beyond Borders: Facilitating the Well-Being of Accompanying Expatriate Partners*	The objective for the current study was firstly to ascertain why accompanying expatriate partners (AEPs) thought strengths of Gratitude, Curiosity and Hope featured so prominently in the model. Secondly, the study aimed to determine how these participants would, from their experience in working with AEPs, enhance these strengths and AEPs' resilience in therapy, and ultimately facilitate greater wellbeing and successful adjustment abroad.	693	1
9	Pienaar et al.	*Peer Helpers' Experience of Participation in an Adventure-Based Experiential Learning Program: A Grit Perspective*	The aim of this study was to explore and describe a group of peer helpers' subjective experiences of their participation in an adventure-based experiential learning program, with a focus on how these experiences related to the concept of grit.	687	0

## If PPIs work in non-weird contexts

This section contains articles investigating the effectiveness of traditional PPIs in non-WEIRD and diverse populations, including under-served, disadvantaged, cross-cultural, or multicultural groups of people. Articles in this section provide theoretical perspectives supporting or critically evaluating relevant positive psychological theories, methods, concepts and constructs underpinning traditional PPI approaches. In total, this section contains four articles, each of which is designed to evaluate the efficacy of specific PPIs or PPI-related approaches and oriented toward how such approaches positively impact specific outcomes in diverse samples of non-WEIRD groups.

In the first article, Donaldson et al. employed the exemplar method to determine whether PPIs positively impact wellbeing. A secondary goal was to use the same approach to identify the most promising method by which PPIs promote wellbeing in WEIRD and non-WEIRD groups. Twenty-five meta-analyses and 42 content review papers were included in the investigation. Of the papers reviewed, only 23-high-quality studies were identified. Within these studies, PPIs positively affected wellbeing outcomes, with most papers indicating a small to moderate effect size. Effect size estimates varied based on different sets of moderating factors, including program format, program type, program duration, age/gender/clinical status of participants, and country in which the study took place. Relevant to the aims of this edited series, larger effect sizes were noted for studies conducted in non-Western countries. However, non-Western studies were also more likely to be rated as lower in design quality. As a secondary component, the research team also identified 14 promising PPIs through an evaluation of the highest quality RCTs. From these 14 intervention, 4 were considered exemplar and deemed most fruitful to be employed with diverse individuals and communities during the global pandemic. These 4 exemplar PPI programs were all multi-component in nature (often addressing strengths, gratitude, positive relationships, positive emotions, and mindfulness) and produced moderate to large changes in wellbeing metrics. Moreover, 75% of the exemplar programs produced these effects with vulnerable and under-served populations. In conclusion, Donaldson et al. asserted PPIs are effective in producing positive changes in wellbeing indices. They also provided unique clinical insights into how wellbeing can be enhanced through different professional services (e.g., psychotherapy). Finally, the research team addressed methodological flaws in RCTs examining the effectiveness of PPIs in non-WEIRD contexts and furnished a set of guidelines to help researchers determine how multi-component PPIs can be adapted to fit the identities, values, and lived experiences among individuals and communities residing in non-WEIRD countries.

Next, Beyebach et al. examined the development of Solution Focused Brief Therapy (SFBT) outcome research and cross-national trends in the production of such research. SFBT is a cost-efficient and effective model employed to resolve interactional problems. The approach relies heavily on helping individuals and groups access and use culturally salient strengths and resources to overcome obstacles in problem resolution, thus, making it a highly compatible approach to PPIs. Using a bibliometric methodology, the research team extracted 365 articles from 12 WEIRD and 21 non-WEIRD countries and determined geographic differences in the production of SFBT outcome science. Results highlighted SFBT as a global approach; research supports its effectiveness cross-culturally and with diverse populations. In recent years, non-WEIRD countries are producing double the amount of SFBT outcome research when compared to WEIRD countries. In addition, non-WEIRD countries are engaging in more RCTs compared to WEIRD countries (63% compared to 37%). These results are encouraging and suggest strength-based models and positive psychological principles are advancing in under-served and cross-cultural contexts. The research team concludes the article by outlining culturally relevant directions by which SFBT and other PPIs can be extended to meet the diverse needs of children, communities, and organizational directives in WEIRD and non-WEIRD spaces.

The third study in this section evaluated whether a multicomponent PPI could positively impact different wellbeing indices in a sample of older adults residing in an inpatient unit. Using a quasi-experimental design, Chamorro-Garrido et al. constructed an 11-week intervention focused on building autobiographical memory functioning and increasing access to different psychological strengths, including forgiveness, gratitude, and humor. Participating older adults were randomly assigned to experimental, placebo, and control groups. Results indicate the multicomponent PPI was effective in producing increases in subjective happiness, life satisfaction, and specific facets of wellbeing and decreases in depression from baseline to post-completion. Moreover, gains in these areas were maintained over a 12-month time span. As a result, these findings highlight multicomponent PPIs as an important resource in supporting a higher quality of life for older adults residing in an inpatient unit, which is a particularly under-researched and vulnerable population.

In the final article in this section, Torkhani et al. investigated the effectiveness of two PPI-integrated physical activity programs, Mindfulness and Implementation Intention, in reducing multiple sclerosis (MS) symptoms among Parisian adults enrolled in outpatient healthcare services. Notable and differentiated within-group differences were detected for each PPI-integrated program. For instance, participants randomly assigned to the Integrated Implementation Intention group reported decreases in all three subcomponents (physical, cognitive, psychosocial) of fatigue, whereas individuals assigned to the Integrated Mindfulness group only reported significant decreases in the physical domain of fatigue. These results highlight some differential patterns in how specific PPI-integrated physical activity programs may alleviate MS symptoms. Considering individuals diagnosed with MS represent a severely neglected population within the PPI literature, this study provides the field with a set of unique and integrated pathways by which PPIs can affect different health-based outcomes.

In total, these four articles help answer the question of whether specific PPIs are effective in increasing wellbeing outcomes and minimizing distress-related to different medical based conditions in under-served, under-researched, and different cultural groups. Moreover, each study leveraged findings to produce key insights into how researchers should methodologically evaluate PPIs and frame PPI programs to increase accessibility and utility in applied work with WEIRD and non-WEIRD populations.

## When do PPIs work in non-weird contexts

This second section of articles focus on elucidating the conditions and contexts by which PPIs are effective in producing positive social, psychological, and health-based outcomes. These articles reflect upon the methods, intervention content, circumstance, and cultural contexts considered in making PPIs work for diverse groups of individuals living in different environmental systems. Based on these criteria, each article aims to cultivate a more complete picture of what is required to develop, design, implement, and evaluate PPIs for non-WEIRD individuals, communities, and organizations.

The first article articulates differential effects in how diverse Mindfulness-Based Interventions contribute to changes in mindfulness, subjective wellbeing, and work engagement/performance outcomes in the Spanish industrial (i.e., managers, administrative staff) workforce. Calcagni et al. evaluated the effects of two Mindfulness-Based Interventions, varying on duration (brief vs. standard length) and format (customized vs. standardized), using an experimental design. The results indicate both programs contributed to heighten levels of positive psychological resources and work engagement/performance outcomes and diminished levels of stress. However, differential effects between the two Mindfulness-Based Interventions were also detected on specific outcomes. For instance, lengthier and standardized mindfulness programs produced greater reported gains in specific mindfulness skills (i.e., non-reactivity) when compared to the briefer and customized mindfulness programs. Alternatively, the customized program appeared more effective in increasing reports of environmental mastery, a key facet of wellbeing, when compared to the longer, standardized program. These differential effects highlight the conditions by which diverse “white collar” workers may benefit the most from mindfulness-based programming.

In the next article, Klibert et al. explored an under-researched set of conditions by which PPIs could bolster positive emotional functioning. Notably, they evaluated whether different savoring interventions, practices designed to generate, maintain, and extend positive emotional experiences through mindful appreciation, could increase positive emotions after the experience of a social-evaluative hassle. This line of examination is unique as most research examines the influence of positive emotional upregulation strategies outside the context of stress and daily hassles. Within an experimental design, the research team evaluated the effects of three savoring interventions and two control conditions. Results highlighted some differential effects. Importantly, the savoring through the moment intervention, was especially effective in minimizing reports of stress and increasing positive emotional experiences when compared to the other conditions. These findings are key as they highlight how specific savoring interventions, mindfully acknowledging and extending positive emotions in the present, may be utilized to support positive coping efforts through strength-based processes. The research team concluded by offering insights into how savoring the moment interventions are a part of a larger and more inclusive method of building resilience in adverse circumstances and how such methods can be adapted to help under-served, marginalized, and vulnerable populations residing in non-WEIRD environments.

Uniquely, Basurrah et al. offered a theoretical review regarding how PPIs can be adapted and utilized to support holistic recovery, quality of life, and wellbeing efforts among individuals residing in Arab countries. Currently, the evaluation of PPIs in Arab specific samples is limited and the field of positive psychology is still in its infancy within these associated countries. The authors used relevant literature to support culturally salient pathways by which PPIs can benefit Arab individuals, communities, and organizations. Specifically, the authors highlight some unique conditions by which researchers should develop and evaluate PPIs. Spiritual traditions, interdependent cultural concepts (e.g., collectivistic beliefs), and family values (e.g., honor, loyalty) are a few cultural considerations to be respected in the development of PPI programs for diverse Arab populations. The authors conclude, if these cultural considerations take a formative role in how PPIs are developed, there is great promise in how PPI programs offset mental health stigma, prevent mental health difficulties, and promote wellbeing in Arab nations.

Although PPIs are generally effective in promoting wellbeing and reducing mental health symptoms, the conditions by which they exact these changes are not well-understood, especially in non-WEIRD populations. These studies address this gap by providing insights into how unique compositions of PPIs can be adapted to meet the needs of diverse individuals, communities, and organizations, especially those located in non-WEIRD environments.

## How/why do PPIs work in non-weird contexts

The final section in this edited volume explores *why* PPIs fail in producing positive outcomes in non-WEIRD contexts. Articles enumerate and address how content-related phenomena, methodological factors, and evaluation methods can be adapted to ensure PPIs yield desired results in diverse populations. This section also emphasizes *how* PPIs affect changes in positive states, traits, cognitions, and behaviors for individuals, communities, and organization residing in non-WEIRD areas. Insights obtained from these articles offer opportunities to shape the process by which PPIs are developed and implemented and set up guidelines to support creative and culturally relevant growth in different facets of wellbeing across different professional arenas (e.g., research, clinical work, policy development).

The first study in this section explored strength-based processes of expatriate partners (spouses, dependents, kin) residing in South Africa using a unique methodology. Notably, Botha et al. constructed a multicomponent and mixed-method study to evaluate new models explaining how character strengths (i.e., hope, gratitude, curiosity) and positive emotional processes (i.e., resilience) contribute to wellbeing within this under-studied population. This study represents the final phase of the project and reflects upon cultural competent psychologists' qualitative perspectives regarding the model developed in previous phases of the project and applied methods used to enhance wellbeing. Narratives were coded for specific themes to organize and guide the development of future PPIs for the partners of expatriates. Results produced four primary and eight sub-themes from the data. These themes outlined how expatriate partners cultivate strengths and resilience and how such mechanisms are connected to wellbeing. Moreover, the research team organized themes in a way that provides clear and culturally competent methods of integrating PPI work into different behavioral healthcare services. The process of assessing client context, setting realistic and culturally responsive goals, working through specific PPI structures (conducting interventions through different time frames), and marshaling external support for the use of strengths and resilience are elaborated upon in great depth. Overall, this study provides a unique roadmap by which applied psychological professionals can collaboratively work alongside the partners of expatriates to support their wellbeing.

Next, Pienaar et al. explored the benefits of participating in a peer helper, adventure-based experiential learning program. Specifically, their study evaluated the qualitative perspectives of 26 South African peer helpers, university students who are trained and supervised in providing interpersonal support for their peers, on how participating in an adventure-based experiential learning program promotes self-growth and grit. Participant narratives (daily reflective diaries and recorded focus group interviews) were collected and evaluated through a thematic content analysis. Analysis resulted in three overarching themes: intrapersonal aspects of grit (e.g., courage), interpersonal aspects of grit (e.g., sense of community), and transpersonal aspects of grit (e.g., continuous growth). Based on the totality of the qualitative review, the adventure-based experiential learning program appeared to be useful in helping peer-helpers grow from a strength-based perspective. Importantly, the rich data provided unique perspectives for how peer-helpers enhance different elements of grit and how grit can be connected to larger facets of wellbeing and life satisfaction.

These two pieces of research describe how the mechanisms of PPIs work in non-WEIRD contexts, by constructing a model to explore the associations between constructs or describing the mechanism on various levels, to explain how or why the PPIs work.

## Future research

The articles in this Research Topic aimed to answer questions regarding “If,” “When,” “How,” and “Why” PPIs work in non-WEIRD contexts. Associated articles were positioned across various academic fields embracing a broad range of themes related to positive psychological health and wellbeing of diverse, under-studied, and vulnerable samples of individuals residing in different parts of the world. Overall, works associated with this Research Topic clearly indicate PPIs are not exclusively WEIRD. Instead, PPIs can be framed through culturally competent and responsive lenses to support individual, community, and organization wellbeing across numerous non-WEIRD contexts. This collection of review and empirical articles provide meaningful insights, perspectives, and guidelines to support the growth of PPIs in non-WEIRD contexts. Notably, these articles offer unique roadmaps describing how strengths and positive emotional experiences are developed and connect to different facets of wellbeing. Moreover, findings generated through these articles give pertinent details about how current PPIs can be adapted and framed to suit the needs of those residing in non-WEIRD environments.

Despite the advances generated through these studies, more work is needed to grow the field of positive psychology in non-WEIRD spaces. For instance, findings from these studies indicate RCTs from non-WEIRD countries were of moderate or low quality, a problem which clearly needs to be addressed. Associated articles outline unique methodological and content (e.g., expanded sample size, long-term follow-up measurements, use of objective/behavioral measures) recommendations to support better research practices for behavioral scientists in non-WEIRD countries. Moreover, the articles exclaim the need to conduct PPI research in under-served and under-studied communities and organizations, including child protection agencies, gender and sexual identity affirming centers, and institutions serving individuals from lower socioeconomic backgrounds, and how different platforms (e.g., online) can increase access to PPI programs within these communities and organizations. Overall, the studies associated with this edited volume speak to the potential of PPIs to bolster recovery and wellbeing efforts in diverse, non-WEIRD populations. However, the field needs to be intentional about building stronger empirical work and developing culturally responsive programs to move pertinent initiatives forward.

## Author contributions

WD and YL drafted the first version of the editorial. JK and LvZ made substantial contributions to the reformulation of the first draft. All authors provided conceptual input and approved the final draft.

## Conflict of interest

The authors declare that the research was conducted in the absence of any commercial or financial relationships that could be construed as a potential conflict of interest.

## Publisher's note

All claims expressed in this article are solely those of the authors and do not necessarily represent those of their affiliated organizations, or those of the publisher, the editors and the reviewers. Any product that may be evaluated in this article, or claim that may be made by its manufacturer, is not guaranteed or endorsed by the publisher.
